# Chronic XBP1s-mediated activation of the unfolded protein response impairs CD8^+^ T cell anti-tumor immunity

**DOI:** 10.3389/fimmu.2026.1940201

**Published:** 2026-10-02

**Authors:** Keith Alan Nair, Jessica Lee, Olivia Zaleski, Luciano Mazzoccoli, Brian Deng, Chih-Chi Andrew Hu, Stanley Ching-Cheng Huang, Andreas Wieland, Hazem E. Ghoneim, Haitao Wen, Xiaopei Huang, Bei Liu

**Affiliations:** 1 Division of Hematology, Department of Internal Medicine, The Ohio State University Comprehensive Cancer Center, Columbus, OH, United States; 2 The Pelotonia Institute for Immuno-Oncology, The Ohio State University Comprehensive Cancer Center, Columbus, OH, United States; 3 Houston Methodist Neal Cancer Center, Houston Methodist Research Institute, Houston, TX, United States; 4 Department of Microbial Infection and Immunity, The Ohio State University College of Medicine, Columbus, OH, United States; 5 Department of Otolaryngology-Head and Neck Surgery, The Ohio State University College of Medicine, Columbus, OH, United States

**Keywords:** anti-tumor immunity, CD8+ T cells, T cell exhaustion, unfolded protein response, XBP1s

## Abstract

CD8^+^ T cells play a crucial role in anti-tumor immunity; however, their functionality is compromised by exhaustion driven by chronic antigen exposure, poor tumor microenvironment, and metabolic dysfunction. Activated CD8^+^ T cells undergo extensive proteomic remodeling, which includes the induction of the IRE1-XBP1 signaling axis of the unfolded protein response. However, the functional consequences of sustained IRE1-XBP1 activation in exhausted CD8^+^ T cells remain elusive. Using a model of constitutive XBP1 activation in CD8^+^ T cells, we demonstrate that sustained XBP1s signaling leads to reduced homeostatic CD8^+^ T cell populations, impaired migratory capacity, and dampened anti-tumor immunity. Notably, pharmacological inhibition of the IRE1-XBP1 axis significantly reduces tumor burden through a dual mechanism that targets both tumor cells and the immune compartment. Our findings reveal a previously unappreciated role for XBP1s in regulating CD8^+^ T cell biology and highlight the IRE1-XBP1 axis as a therapeutic target in solid tumors.

## 1 Introduction

CD8^+^ T cells are central effectors of tumor control and anti-tumor immunity. However, strategies to improve their functionality remain an active area of investigation (1–6). Upon activation, CD8^+^ T cells undergo extensive proteomic remodeling, resulting in a three- to fourfold increase in total cellular protein content (7). This heightened biosynthetic demand necessitates tightly regulated protein quality control mechanisms to maintain protein homeostasis, including activation of the unfolded protein response (UPR). The UPR is comprised of three principal signaling branches —inositol-requiring enzyme 1 (IRE1), protein kinase RNA-like endoplasmic reticulum kinase (PERK), and activating transcription factor 6 (ATF6), which have gained increasing attention for their roles in regulating proteostasis and cellular metabolism (8–10). Under homeostatic conditions, these pathways are maintained in an inactive state through association with the endoplasmic reticulum (ER) chaperone BiP, which suppresses their activity (9). Upon activation, the three UPR branches initiate both distinct and overlapping signaling programs that coordinate cellular adaptation to proteotoxic stress.

Among the UPR signaling arms, the IRE1 pathway regulates two principal processes: regulated IRE1-dependent decay (RIDD) of select mRNAs and the unconventional splicing of X-box binding protein 1 (XBP1u) mRNA to generate the active transcription factor XBP1s. Both processes are mediated by the endoribonuclease activity of IRE1 and regulate transcriptional and post-transcriptional programs that influence cellular metabolism, survival, and effector differentiation (8, 9, 11). Given the sustained antigen exposure and metabolic stress characteristic of the tumor microenvironment, IRE1/XBP1 signaling is poised to modulate CD8^+^ T cell functional states, including exhaustion, with potential implications for anti-tumor immunity and immunotherapeutic efficacy.

Studies examining the role of the UPR in CD8^+^ T cells remain limited. Previous studies reported that loss of IRE1α or XBP1s does not impair CD8^+^ T cell development (12) or alter CD8^+^ T cell frequency (13), respectively. Subsequent studies showed that XBP1s expression is induced in CD8^+^ T cells following CD3 and CD28 ligation in the presence of IL-2 (14), and that cholesterol accumulation in the tumor microenvironment (TME) promotes XBP1s upregulation, leading to increased PD-1 expression (15). In human multiple myeloma patient samples, elevated XBP1s levels in CD8^+^ T cells were further linked to reduced glutamine uptake through downregulation of the glutamine transporter, SNAT2 (16). Despite these observations, the broader functional consequences of sustained UPR activation in CD8^+^ T cells, particularly within the TME, remain poorly defined (17–19).

To address these gaps and directly interrogate the impact of persistent XBP1s signaling, we generated a novel mouse model that constitutively overexpresses XBP1s specifically in CD8^+^ T cells. We demonstrate that persistent XBP1s signaling impairs T cell homeostasis, significantly reducing peripheral CD8^+^ T cell numbers. In a tumor model, persistent XBP1s signaling leads to increased tumor burden and diminished effector function. Mechanistically, we link these deficits to a downregulation of the key chemokine receptor CXCR3, which compromises T cell migratory capacity. Finally, we show that therapeutic inhibition of the IRE1α/XBP1s axis using the inhibitor B-I09 suppresses tumor growth and favorably reshapes the differentiation state of tumor-infiltrating CD8^+^ T cells.

## 2 Materials and methods

### 2.1 Mice

All animal experimental protocols were approved by The Ohio State University (OSU) Institutional Animal Care and Use Committee (IACUC) (Protocol number: 2020A00000015). All methods were carried out in accordance with federal regulations and established institutional guidelines. CD8^+^ T cell-specific XBP1s-overexpressing mice (Rosa26-LSL-XBP1s^+/+^CD8Cre^+^) and control littermates (Rosa26-LSL-XBP1s^+/+^CD8Cre^-^) were generated by crossing Rosa26-LSL-XBP1s mice with E8_I_-CD8Cre (The Jackson Laboratory, stock no. 008766). OT-I-specific XBP1s overexpression mice were generated by crossing our Rosa26-LSL-XBP1s^+/+^CD8Cre^+^ with OT-I mice (The Jackson Laboratory, stock no. 003831). Mice used for experiments were either female or male aged 6–12 weeks. All mice were maintained on the C57BL/6 background and housed at The Ohio State University under IACUC-approved guidelines.

### 2.2 Reagents, cell lines, and software

Antibodies used in this study were acquired from eBioscience, Life Technologies, BD Biosciences, BioLegend, Thermo Fisher Scientific, and Cell Signaling Technologies. Percoll was purchased from Cytiva Life Sciences. Dispase was acquired from Worthington Biochemical Corporation. Other media and chemicals were obtained from Sigma-Aldrich and Thermo Fisher Scientific. B-I09 (an IRE1 RNase inhibitor) was developed and generously provided by Dr. Chih-Chi Andrew Hu at the Houston Methodist Academic Institute. Cancer cell lines have been maintained by the Liu Laboratory. Software used in this study includes: SpectroFlo from Cytek BioSciences, FlowJo from BD Life Sciences, and GraphPad Prism from GraphPad Software.

### 2.3 Analysis of human patient data

Single-cell analysis was performed using the Tumor Immune Single-cell Hub 2 (TISCH2) software accessed on November 6^th^, 2024, via the URL https://tisch.compbio.cn/home/. The dataset analyzed was CRC_GSE108989 in the ‘Dataset’ tab at the top of the page. In the middle of the page, the ‘Gene’ tab was clicked, and *XBP1* was selected. The population selection was ‘Celltype (minor-lineage)’. For the gene correlation data, the ‘Gene’ tab at the top of the page was selected. *XBP1* was entered as the gene, the cell-type annotation was ‘Celltype (minor-lineage)’, the dataset included was ‘CRC (Colorectal Cancer)’, the cell type included was ‘CD8T (CD8 T cells)’, and the lineage for calculating correlation was ‘CDT’. The selected data was CRC_GSE108989.

### 2.4 Flow cytometry

Spleen and lymph nodes were mechanically disassociated through a 40 μm strainer. Red blood lysis was performed on splenocytes with ACK lysis buffer (0.15M NH_4_Cl, 0.01M KHCO_3_, 0.1mM EDTA). Tumor-infiltrating lymphocytes (TILs) were isolated after digestion with collagenase D (2 mg/mL, Sigma-Aldrich), DNase I (100 μg/mL, Sigma-Aldrich), and Dispase (0.075 μg/mL, Worthington BioChemical Corporation). A 40%/80% Percoll gradient was performed to isolate single cells after digestion (Cytiva). For staining cell surface markers, cells were stained with viability dye for 10 minutes, FcR blocking antibodies for 10 minutes, and the cell surface antibody cocktail for 30 minutes on ice. If the antibody cocktail contained antibodies for chemokine receptors, samples were stained for 20 minutes at room temperature. For intracellular staining, cells were fixed and permeabilized with the eBioscience Foxp3/Transcription Factor kit (Invitrogen) after cell surface staining. To stain for cytokines, cells were treated with PMA (50 ng/mL), Ionomycin (500 ng/mL), and Brefeldin A (3 μg/mL) for three hours before staining. Data were collected using the Cytek Aurora flow cytometer (Cytek Biosciences) and analyzed with FlowJo version 10 (BD Life Sciences). High-dimensional analysis was performed using the web-based software OMIQ. After gating, cells were visualized using the UMAP algorithm (48) and then clustering analysis was performed with the FlowSOM (49, 50) algorithm. Afterwards, a heatmap was applied to analyze broad patterns.

### 2.5 *In vitro* CD8^+^ T cell activation

Spleen and lymph nodes were isolated as described above. Afterward, negative selection was performed on the samples via the mouse naïve CD8a^+^ T cell isolation kit (Miltenyi Biotec). Cells were then stained with CellTrace Violet (Thermo Fisher) and incubated with mouse CD3/CD28 DynaBeads (Thermo Fisher) at a ratio of 1 bead to 1 cell and 50 U/mL of rhIL-2 (Thermo Fisher) for four days at 37°C, 5% CO_2_ in T cell media. After two days, half of the media was removed and refreshed with new media and additional rhIL-2.

### 2.6 RNA sequencing

CD8^+^ T Cells were sorted into the required populations by FACS using the Cytek Aurora CS System. After sorting, cells were snap frozen and stored at -80 °C. Samples were then sent to Azenta Life Sciences (New Jersey) for bulk RNA-seq preparation and analysis. Briefly, the cell pellet RNA was extracted by Azenta Life Sciences, and the mRNA was selected based on the poly(A) tail. The library was prepared and run using the HiSeq platform with a 2x150 bp configuration. Original read counts were normalized and then used to determine the differentially expressed genes. DESeq2 was used to perform the comparison, with the Wald test conducted to generate p-values and log2 fold changes. A gene was considered differentially expressed if the adjusted p-value was less than 0.05 and the log2 fold change was greater than or less than ±1, respectively. Gene ontology analysis was performed by clustering the significantly differentially expressed genes according to their gene ontology, and then running GeneSCF with Fisher’s Exact Test (GeneSCF v1.1-p2).

### 2.7 Tumor studies

In the MC38 studies, 1 x 10^6^ cells were injected subcutaneously on the lower right flank in 100 μL of sterile saline. Tumor size was measured (length x width) every two days for two weeks, and afterward was measured daily using a digital caliper. Tumor volume was calculated using (length^2^ x width) x ½. For IRE1 α/XBP1s inhibitor treatment, we inoculated 1 x 10^6^ MC38 cells. When the tumor size reached 35 mm^3^ on day 7, B-I09 or vehicle control (DMSO) was administered at 50 mg/kg intraperitoneally 5 days per week for two weeks. Mice were monitored every two days for tumor growth.

### 2.8 Migration assay

This protocol was adapted from Barreira da Silva et al. (40) Nat Immunol (40, 41). Briefly, OT-I and OT-I-XBP1s-OE spleens were processed as described above into single-cell suspensions. Cells were then resuspended in 10 mL of TCM containing 1 g/mL SIINFEKL for 1 hour at room temperature on a shaker. Cells were washed and plated with 50 U/mL of rhIL-2, then placed in an incubator set to 37 °C with 5% CO_2_. The media was refreshed every two days by removing the top half and replacing it with fresh media and rhIL-2. After six days, the Transwell plate was prepared by adding the diluted recombinant murine CXCL10 (Thermo Fisher) to the lower wells, and 5 x 10^5^ cells were added to the upper wells in triplicate. The plate was incubated for 2 hours at 37 °C with 5% CO_2_ and then prepared for flow cytometry. Counting beads were used according to the manufacturer’s protocol (Thermo Fisher, catalog #C36950).

### 2.9 Statistical analysis

Statistical analysis was performed with Prism 9 (GraphPad). Data points are biological replicates that are presented as the mean with SEM. Tumor growth curves were analyzed via a 2-way ANOVA. Pairwise comparisons were analyzed with a two-tailed unpaired Student’s *t*-test. A p-value of <0.05 was considered statistically significant.

## 3 Results

### 3.1 Activated CD8^+^ T cells exhibit elevated XBP1s expression in human and murine tumors

Given the limited characterization of XBP1s expression in CD8^+^ T cells in prior studies, we first sought to determine whether XBP1s is upregulated in human tumor-infiltrating CD8^+^ T cell populations. To this end, we analyzed a publicly available single-cell RNA-sequencing dataset from TISCH2 (20, 21). Analysis of colorectal cancer samples revealed that *XBP1* expression was enriched predominantly within subsets of activated T cells (**Figures 1A, B**). We also identified the top 15 genes that were significantly associated with *XBP1* expression in tumor-infiltrating CD8^+^ T cells, including genes involved in cytoskeletal organization and cell membrane dynamics (*ANXA, PXN, DSTN*) as well as UPR-related genes (*FKBP11, HOPX*) (**Figure 1C**). By stratifying CD8^+^ T cells into central memory (T_cm_), effector (T_eff_), and exhausted (T_ex_) subsets, we observed increased *XBP1* expression across all populations, with the highest expression observed in the T_eff_ cells. It has been shown previously that T cell receptor (TCR) stimulation in the presence of IL-2 induces XBP1 splicing into XBP1s (14). To assess the activation of the UPR in murine CD8^+^ T cells following *in vitro* and *in vivo* activation, CD8^+^ T cells were stimulated with CD3/CD28 DynaBeads *in vitro* or isolated from MC38 tumors *in vivo*, respectively. In both instances, it was found that the percentage of XBP1s-expressing CD8^+^ T cells was significantly increased, as assessed by spectral flow cytometry (**Figures 1E, F**). Collectively, these data demonstrate that XBP1s expression is induced in activated CD8^+^ T cells across both human and murine tumor contexts, raising the possibility that sustained XBP1s signaling may influence CD8^+^ T cell maintenance and function.

**Figure 1:**
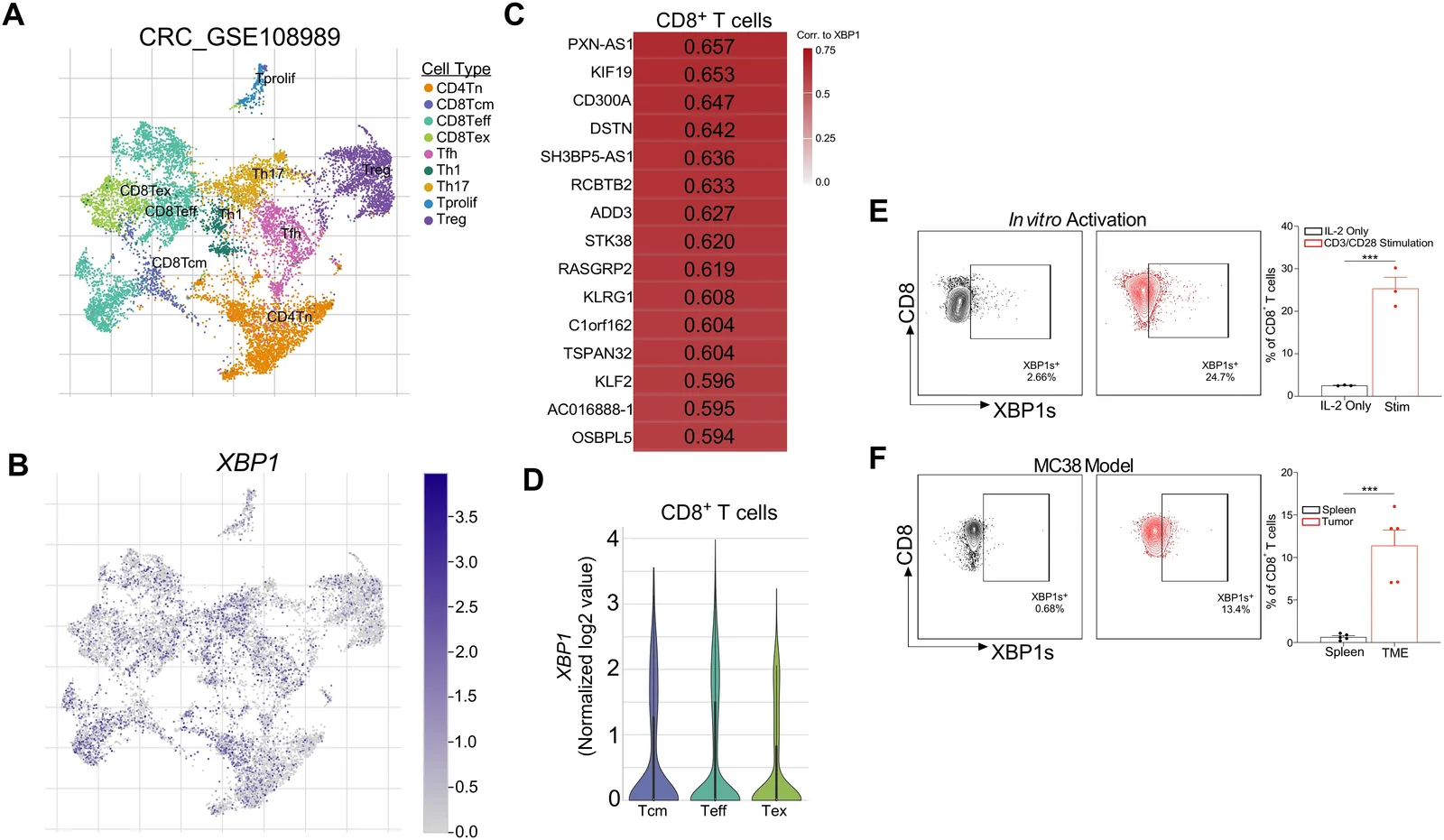
XBP1s is upregulated in activated human and murine CD8^+^ T cells. **(A)** UMAP visualization with annotated cell clusters from human colorectal cancer samples generated using the TISCH2 platform. **(B)** Feature plot showing XBP1 expression overlaid on the UMAP from the same dataset. **(C)** Heatmap of the top 15 genes significantly correlated with *XBP1* expression in human CD8^+^ T cells. **(D)** Violin plot depicting normalized log_2_ *XBP1* expression across central memory (Tcm), effector (Teff), and exhausted (Tex) CD8^+^ T cell subsets. **(E)** Murine control CD8^+^ T cells were activated *in vitro* for four days with either IL-2 only or anti-CD3/CD28 DynaBeads plus IL-2. Representative flow plots are provided along with quantification (n = 3 per group). **(F)** Control mice were inoculated with 1 x 10^6^ cells on the right flank subcutaneously and allowed to grow for 21 days. Spleens and tumors were harvested and analyzed by flow cytometry. Representative plots and quantification are shown (n = 5 per group). *P < 0.05, **P < 0.01, ***P < 0.001, ****P < 0.0001. (Quantifications were analyzed by a 2-tailed unpaired *t*-test).

### 3.2 XBP1s stress signaling impairs peripheral CD8^+^ T cell homeostasis and upregulates the UPR

Given that XBP1s is upregulated in CD8^+^ T cells after activation and within the TME, we next directly assessed the cell-intrinsic effects of chronic XBP1s signaling on CD8^+^ T cell homeostasis. To investigate this, we generated a CD8^+^ T cell–specific XBP1s overexpression model using the Rosa26-LSL-Cas9 and E8_I_-CD8Cre system. In this model, Cre recombinase expression is driven by the E8_I_ enhancer of the *Cd8* locus, enabling selective genetic manipulation in single-positive CD8^+^ thymocytes and their progeny (22). Following CD8 upregulation during thymic development, Cre-mediated recombination excises the loxP-flanked stop cassette, permitting targeted gene expression specifically in CD8^+^ T cells. Utilizing CRISPR/Cas9, an XBP1s construct tagged with eGFP was inserted downstream of the loxP-stop-loxP (LSL) cassette, allowing direct tracking of XBP1s expression (**Figure 2A**). Flow cytometric analysis of splenocytes confirmed eGFP expression exclusively within the CD8^+^ T cell compartment, validating the specificity of the model (**Figure 2B**). To further confirm XBP1s protein overexpression, intracellular staining was performed and confirmed that XBP1s is overexpressed (**Figure 2C**). We then characterized the effects of XBP1s overexpression in developing CD8^+^ T cells by examination of thymic populations. Flow cytometric analysis revealed no significant differences in the frequency or absolute number of thymocyte subsets between control and XBP1s-overexpressing (XBP1s-OE) mice (**Supplementary Figure S1**), indicating that XBP1s overexpression does not impair CD8^+^ T cell development. In contrast, analysis of peripheral lymphoid tissues demonstrated a significant reduction in both the frequency and absolute number of CD8^+^ T cells in the spleen (**Figure 2D**), with a similar trend observed in the peripheral (inguinal) lymph nodes (LNs) (**Supplementary Figure S2**). The corresponding increase in CD4^+^ T cell frequency did not reach statistical significance. Given that XBP1s overexpression is restricted to the CD8^+^ compartment under the E8i-CD8 Cre driver, the phenotypes reported here are unlikely to reflect a CD4^+^ T cell-intrinsic effect of XBP1s.

**Figure 2:**
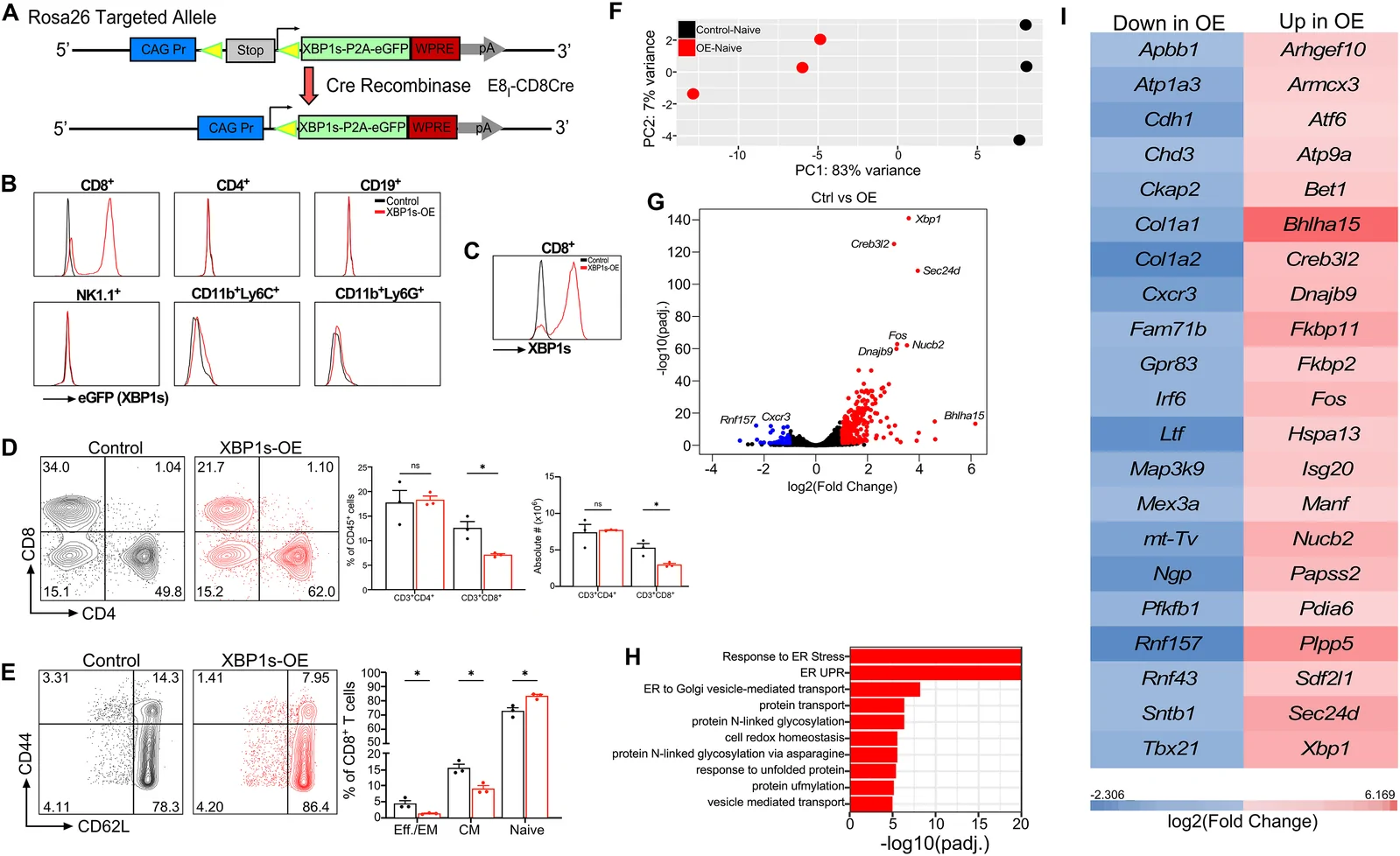
CD8^+^ T cell-specific XBP1s overexpression leads to reduced peripheral CD8^+^ T cell abundance and unfolded protein response upregulation. **(A)** Schematic of the genetic strategy used to conditionally express XBP1s in CD8^+^ T cells using the Rosa26-LoxP-STOP-LoxP system and E8_I_-CD8Cre model. **(B)** Representative flow plots showing eGFP reporter expression across multiple immune cell populations. **(C)** Intracellular staining for XBP1s in total CD8^+^ T cells from splenocytes. **(D)** Representative flow cytometry analysis of splenocytes from control and XBP1s-OE mice. Flow cytometry data are gated as follows: FSC-A/SSC-A, Live/Dead negative, CD45^+^, CD3^+^. Quantification is shown. Absolute numbers of CD4^+^ and CD8^+^ T cells in the spleen, calculated by multiplying subset frequencies by total viable cell counts determined by Trypan Blue exclusion (n = 3 per group). **(E)** Representative flow plots of CD8^+^ T cell subsets from control and XBP1s-OE mice. Naïve cells were defined as CD44^-^CD62L^+^, effector/effector memory cells as CD44^+^CD62L^-^, and central memory (Tcm) cells as CD44^+^CD62L^+^. Flow cytometry data is gated as follows: FSC-A/SSC-A, Live/Dead negative, CD45^+^, CD3^+^, CD8^+^, eGFP^-^ or eGFP^+^. **(F)** Naïve CD8^+^ T cells were sorted by spectral flow cytometry using the following gating strategy: FSC-A/SSC-A, Live/Dead negative, CD45^+^, CD3^+^, CD8^+^, CD62L^+^/CD44^-^, eGFP^+^ or eGFP^-^. RNA sequencing was performed by Azenta Life Sciences. Principal component analysis (PCA) illustrates transcriptional separation between control and XBP1s-OE naïve CD8^+^ T cells. **(G)** Volcano plot depicting differentially expressed genes, with transcripts upregulated in XBP1s-OE cells shown in red and downregulated genes shown in blue. **(H)** Gene ontology analysis showing the ten most significantly upregulated pathways in XBP1s-OE CD8^+^ T cells. **(I)** Selected differentially expressed genes displayed as log_2_(fold change) values in XBP1s-OE cells, listed alphabetically. Data are presented as mean ± SEM. *P < 0.05, **P < 0.01, ***P < 0.001, ****P < 0.0001; statistical significance was determined by two-tailed unpaired *t* tests.

To further assess the impact of XBP1s overexpression on CD8^+^ T cell activation, we evaluated expression of CD44 and CD62L, markers commonly used to define naive, effector, and central memory subsets (23). XBP1s-OE mice exhibited a reduction in effector/effector memory (CD44^+^/CD62L^-^, T_eff/em_) and central memory (CD44^+^/CD62L^+^, T_cm_) CD8^+^ T cells, accompanied by a relative increase in the naïve (CD44^-^/CD62L^+^, T_n_) population (**Figure 2E**). To define the transcriptional consequences of sustained XBP1s signaling, we sorted T_n_ CD8^+^ T cells by spectral flow cytometric sorting and performed RNA sequencing. We performed principal component analysis (PCA) and found a large difference between the control and XBP1s-OE CD8^+^ T cells (**Figure 2F**). Specifically, genes such as *Creb3l2*, *Nucb2*, and *Dnajb9* were upregulated, confirming sustained UPR activation (**Figure 2G**). Gene ontology analysis demonstrated enrichment of UPR- and protein transport-associated pathways in the XBP1s-OE cells (**Figure 2H**). Interestingly, we also found that the chemokine receptor *Cxcr3* was significantly downregulated in the XBP1s-OE CD8^+^ T cells (**Figures 2G, I**). Together, these findings indicate that constitutive XBP1s signaling disrupts both CD8^+^ T cell homeostasis and fate.

### 3.3 Constitutive XBP1s upregulation reduces CD8^+^ T cell engagement and effector function within the TME

Given that XBP1s is induced in activated CD8^+^ T cells within the TME and that constitutive XBP1s signaling disrupts peripheral CD8^+^ T cell homeostasis, we next examined how chronic ER stress influences anti-tumor immunity *in vivo*. MC38 murine colon carcinoma cells were implanted subcutaneously into control and XBP1s-OE mice. We found that XBP1s-OE mice developed significantly larger tumor than controls (**Figure 3A**). Analysis of tumors and draining lymph nodes (dLNs) by spectral flow cytometry revealed a significant reduction in both the frequency and absolute number of CD8^+^ T cells in the TME and dLNs of XBP1s-OE mice relative to controls (**Figure 3B**). Phenotypic characterization of CD8^+^ TILs demonstrated a significant decrease in T_eff/em_ cells, with corresponding increases in both T_cm_ and T_n_ cells (**Figure 3C**). We focused this analysis on the CD44^+^CD62L^-^ (effector/effector memory) subset, as this population represents the dominant tumor-engaged compartment and exhibited the largest shift in frequency with XBP1s overexpression (**Figure 3C**). Further examination of the T_eff/em_ compartment revealed decreased PD-1 and TIM-3 co-expression in XBP1s-OE CD8^+^ T cells, suggesting diminished tumor engagement and chronic stimulation, that is consistent with their diminished effector functionality and reduced TME infiltration (**Figure 3D**). Functionally, XBP1s-OE CD8^+^ T cells exhibited significantly decreased production of IFN-γ and TNF-α production following three hours of *ex vivo* stimulation with PMA and Ionomycin (**Figure 3E**). We also found that XBP1s-OE CD8^+^ T cells had significantly reduced levels of the effector-related transcription factor Tbet and decreased effector cytokine granzyme B (**Supplementary Figure S3**). Unlike the CD44^+^CD62L^-^ (effector/effector memory) subset, we found that PD-1 and TIM-3 expression levels in the CD44^+^CD62L^+^ and CD44^-^CD62L^+^ CD8^+^ T cell subsets are comparable between control and XBP1s-OE CD8^+^ T cells (**Supplementary Figure S4**). Together, these data indicate that sustained XBP1s expression attenuates CD8^+^ T cell activation, effector differentiation, and cytokine production within the TME, thereby compromising anti-tumor immune responses.

**Figure 3:**
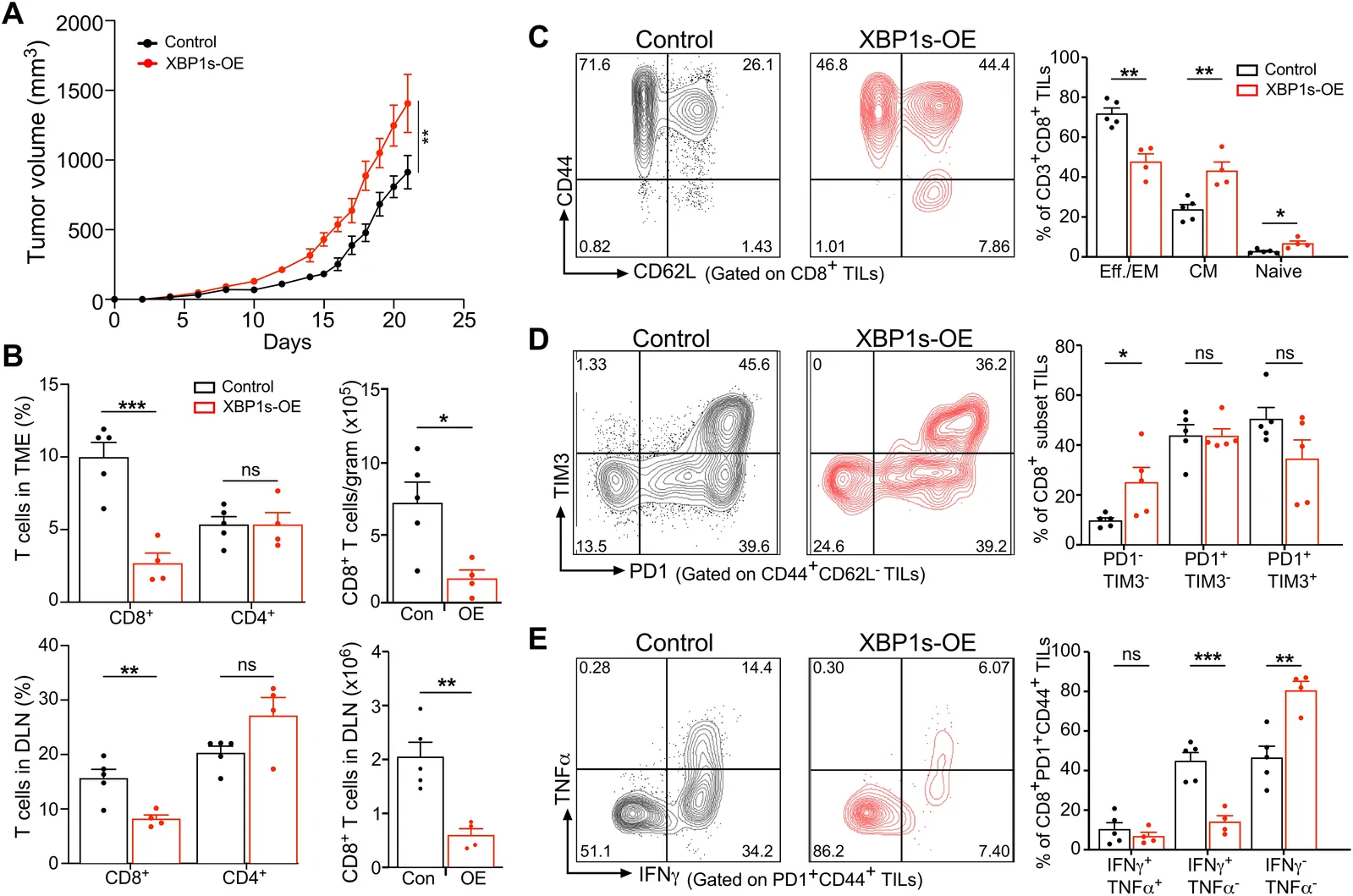
CD8^+^ T cell-specific XBP1s expression impairs anti-tumor immunity. **(A)** MC38 tumor growth kinetics in control or XBP1s-OE mice (n = 9–10 per group). **(B)** Frequency and absolute number of live CD8^+^ T cells in the TME and tumor-dLN, with representative quantification shown (n = 4–5 per group). **(C)** Representative flow cytometry plots and quantification of CD62L and CD44 expression on CD8^+^ T cells (n = 4–5 per group). **(D)** Representative flow plots and quantification of PD-1 and TIM3 expression on effector/effector memory CD8^+^ T cells (CD44^+^ CD62L^-^) isolated from tumors (n = 4–5 per group). **(E)** Representative flow cytometry plots and quantification of IFN-γ and TNF-α production by tumor-infiltrating CD44^+^ PD-1^+^ CD8^+^ T cells following 3 hours of PMA and ionomycin stimulation (n = 4–5 per group). Data are presented as mean ± SEM. *P < 0.05, **P < 0.01, ***P < 0.001, ****P < 0.0001. (Tumor volume in **(A)** was analyzed by 2-way ANOVA; all other comparisons were analyzed using two-tailed unpaired *t* tests.

### 3.4 Chronic XBP1s activation limits CXCR3 expression and migration capacity in CD8^+^ T cells

Our data showed that chronic XBP1s expression altered CD8^+^ T cell homeostasis and impaired tumor control; thus, we next investigated whether sustained UPR activation intrinsically reprograms CD8^+^ T cells’ migratory capability as well. CXCR3 is a chemokine receptor associated with recruitment to inflamed tissues, promoting an effector phenotype, and is essential for the bystander activation of CD8^+^ T cells (24, 25). To determine whether reduced *Cxcr3* transcription corresponded to diminished surface expression (**Figure 2I**), we performed flow cytometric analysis of CD8^+^ T cells isolated from the spleens and inguinal lymph nodes (iLNs) of control and XBP1s-OE mice. Consistent with the RNA sequencing data, XBP1s-OE CD8^+^ T cells exhibited significantly lower CXCR3 protein expression under homeostatic conditions in both spleens and ILNs (**Figure 4A**). After *in vitro* activation, this same phenomenon was present, and was not dependent on differentiation status, with both T_eff_ and T_cm_ CD8^+^ T cells displaying significantly decreased CXCR3 expression in the XBP1s-OE cells (**Figure 4B**). This suggests that CD8^+^ T cells with sustained UPR upregulation exhibit impaired migratory capabilities. CXCR3 is primarily expressed on activated CD8^+^ T cells, among other cell types, and mediates migration in response to its cognate chemokines CXCL9 or CXCL10 (26). It has also been shown to be highly expressed in inflamed tissues on CD8^+^ T cells (27). Several studies have shown that the infiltration and migration of CD8^+^ T cells in murine and human tumors depend on CXCR3 expression (28–37), which is partially regulated by T-bet (38).

**Figure 4:**
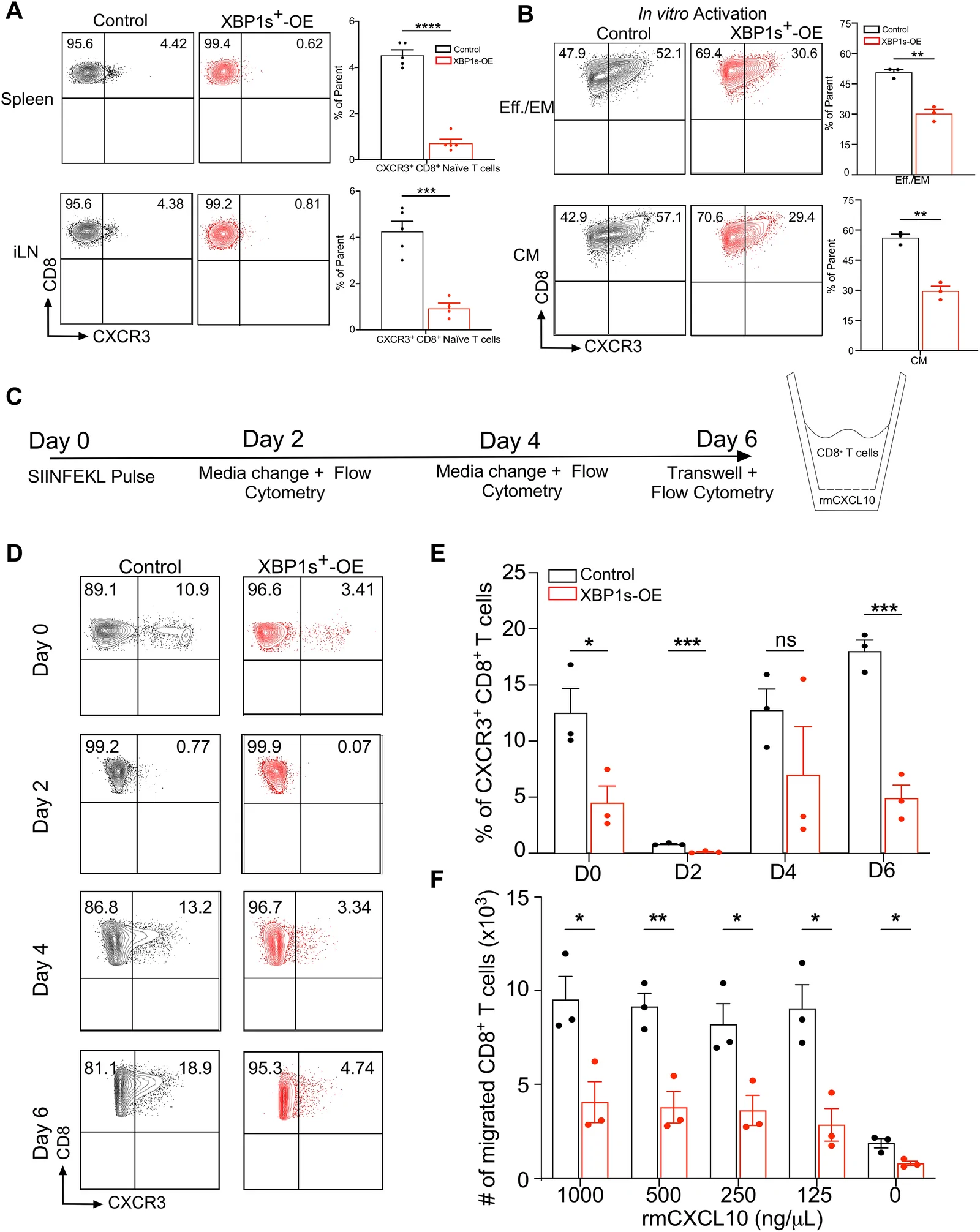
Chronic XBP1s signaling impairs CD8^+^ T cell migration via CXCR3 downregulation. **(A)** Representative flow cytometry plots and quantification of CXCR3 expression on naïve (CD62L^+^/CD44^-^) CD8^+^ T cells isolated from the spleen and inguinal lymph nodes (iLN) of steady-state mice. **(B)** Representative flow cytometry plots and quantification of CXCR3 expression on *in vitro* activated CD8^+^ T cells, comparing effector/effector memory (CD62L^-^/CD44^+^) and central memory (CD62L^+^/CD44^+^) populations. Flow cytometry data represents n = 3–5 mice per group **(C)** Schematic of the *in vitro* activation and migration assay timeline. **(D)** Representative flow cytometry plots of CXCR3 expression on CD8^+^ T cells after 0, 2, 4, and 6 days of *in vitro* activation and expansion. **(E)** Quantification of the percentage of CXCR3^+^ CD8^+^ T cells from **(D)**. **(F)** Transwell migration of activated CD8^+^ T cells toward the CXCR3 ligand CXCL10. n = 3 per group. Data are shown as mean ± SEM. *P < 0.05, **P < 0.01, ***P < 0.001, ****P < 0.0001. Statistical significance was determined by a two-tailed unpaired *t*-test.

To determine if XBP1s overexpression similarly reduces migration in activated CD8^+^ T cells, we employed an *in vitro* activation and Transwell migration assay. To perform this, we crossed XBP1s-OE mice with OT-I T cell receptor transgenic mice to generate antigen-specific CD8^+^ T cells constitutively overexpressing XBP1s, which recognizes a short peptide sequence from the ovalbumin (OVA) protein called SIINFEKL (39). Following brief SIINFEKL peptide stimulation and 6 days of *in vitro* expansion (**Figure 4C**), we found that OT-I-XBP1s-OE CD8^+^ T cells exhibited significantly reduced CXCR3 surface expression compared with controls (**Figures 4D, E**). We then took these expanded cells and performed a Transwell migration assay using the CXCR3 ligand CXCL10 (40, 41). We found that XBP1s-OE CD8^+^ T cells had markedly impaired migratory capabilities across all CXCL10 concentrations tested (**Figure 4F**). This finding led us to hypothesize that pharmacological inhibition of the IRE1α-XBP1s pathway might conversely promote T cell recruitment to the TME.

### 3.5 Pharmacologic inhibition of the expression of XBP1s suppresses tumor growth and restores CD8^+^ T cell function

Given the XBP1s-dependent transcriptional and functional defects in CD8^+^ T cell migration and anti-tumor immunity, we next asked whether pharmacologic inhibition of XBP1 splicing could reverse these effects *in vivo*. To assess the therapeutic potential of this pathway, we utilized the novel IRE1α-splicing inhibitor, B-I09, which selectively blocks the ability of IRE1α to splice the *XBP1u* transcript into the *XBP1s* form. B-I09 has previously demonstrated efficacy in mouse models of leukemia, ovarian cancer, and graft-versus-host disease. On-target inhibition of the IRE1α-XBP1 pathway by B-I09 has been directly established in these prior *in vivo* studies, and none reported treatment-related hepatic or renal toxicity at comparable dosing, including a model of chronic graft-versus-host disease in which hepatic pathology was a primary endpoint (42–46), supporting both the on-target activity and favorable safety profile of this inhibitor in our study. However, its effects on CD8^+^ T cell activation, migration, and differentiation in solid tumors have not been investigated. To investigate this, wild-type mice bearing established MC38 tumors were treated with B-I09 (50 mg/kg, five times per week for two weeks), beginning seven days after tumor implantation. B-I09 treatment resulted in a significant reduction in tumor growth compared with vehicle-treated controls (**Figure 5A**). Analysis of the TME and dLNs revealed no significant differences in the overall frequency or absolute number of infiltrating CD4^+^ or CD8^+^ T cells between treatment groups (**Figure 5B**), suggesting that B-I09 does not broadly alter T cell infiltration. However, examination of exhaustion markers revealed there was an increase in the PD-1^intermediate^ population, seemingly due to a decrease in the PD-1^+^/TIM3^+^ cells, indicating a shift away from terminal exhaustion toward a more functionally competent, tumor-engaged population in the TME (**Figure 5C**). Upon further examination of the tumor-reactive CD8^+^ T cell compartment, defined as PD-1^+^CD44^+^ CD8^+^ T cells, we discovered that there is a decrease in the TIM3^+^LAG3^+^ population, representing terminal exhaustion (**Figure 5D**). To visualize treatment-induced changes in the CD8^+^ T cell landscape within the TME, we performed high-dimensional flow cytometry followed by UMAP dimensionality reduction and FlowSOM clustering analysis. From this, we found that cluster 9 was increased in the B-I09-treated group (**Figure 5E**). Heatmap analysis of this cluster showed enrichment for early tumor-reactive activation markers, including CD44, PD-1, CD69, and CXCR3 (**Figure 5F**). Performing a similar analysis of the transcription factors, we found that there were increased clusters related to TCF1 expression, which represents an early-exhaustion phenotype (**Supplementary Figure S5**). Notably, increased CXCR3 expression in this population aligns with our earlier findings that sustained XBP1s signaling represses CXCR3 expression, suggesting that inhibition of IRE1-XBP1s signaling may restore the T_progenitor_ population in the TME (**Figure 5F**).

**Figure 5:**
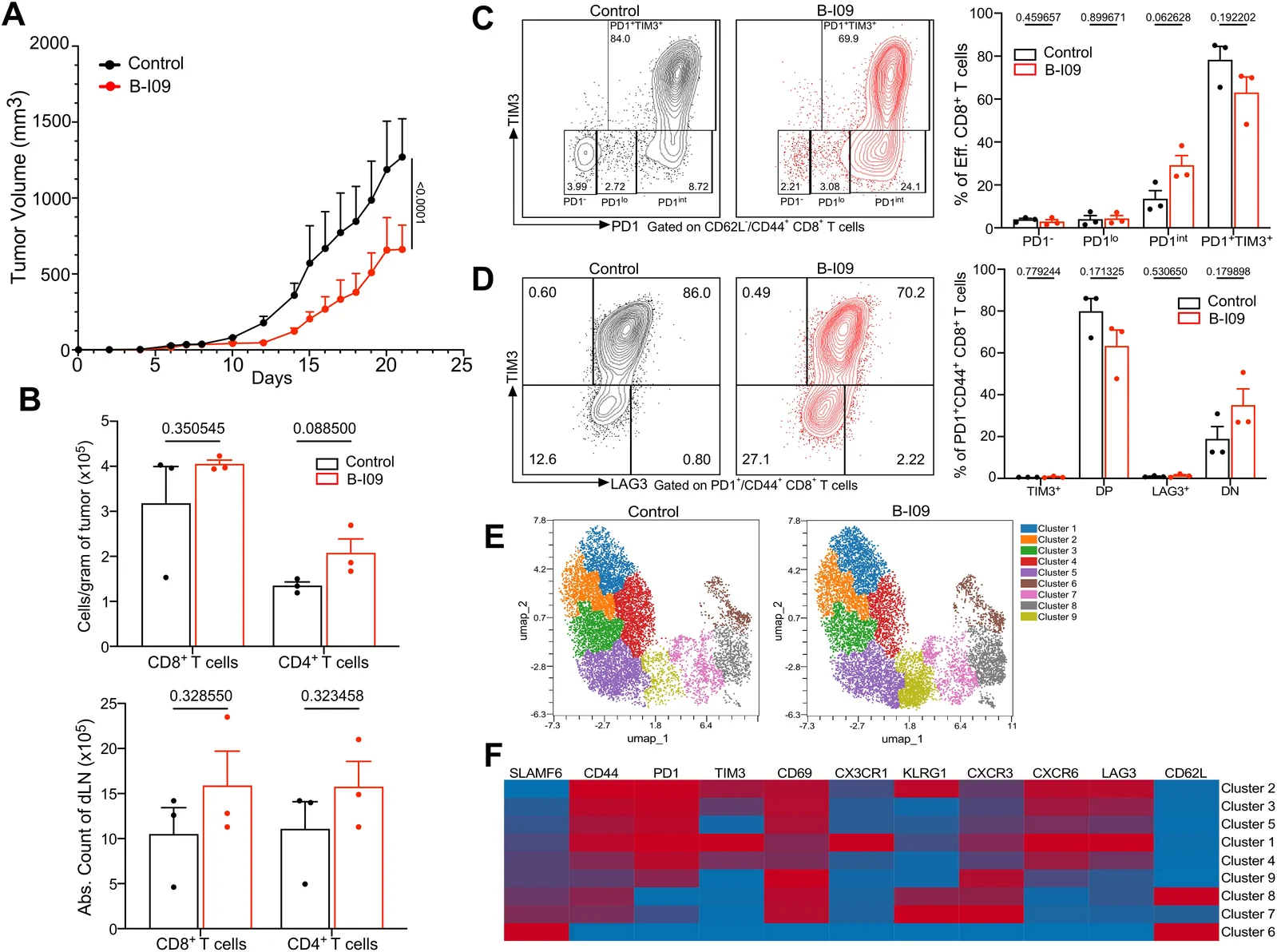
Pharmacologic IRE1/XBP1s inhibition reduces tumor burden and promotes functional CD8^+^ T cell states in the TME. **(A)** MC38 tumor growth curves in vehicle control versus B-I09 treated mice (n = 3 per group). **(B)** Quantification of T cell infiltration into the TME and dLN (n = 3 per group). **(C)** Representative flow cytometry plots showing PD-1 and TIM3 expression on CD44^+^ CD62L^-^ CD8^+^ T cells, with quantification (n = 3 per group). **(D)** Representative flow cytometry plots of LAG3 and TIM3 expression on PD1^+^ CD44^+^ CD8^+^ T cells with quantification provided. n = 3 per group **(E)** Visualization of CD8^+^ T cells in the vehicle control and B-I09 treated mice. Graphs represent concatenated samples. n=3 per group. **(F)** Heatmap showing relative mean fluorescence intensity of marker expression in each cluster from panel **(E)** P values are indicated. Statistical significance was determined by two-way ANOVA **(A)** or two-tailed unpaired *t*-test **(B–D)**.

## 4 Discussion

CD8^+^ T cells are central effectors of anti-tumor immunity and serve as a foundation for many successful cancer immunotherapies. However, their effectiveness within tumors is limited by several factors, including exhaustion, impaired migration, immunosuppressive cytokines and cellular populations, and metabolic dysregulation (2–6). Given the substantial increase in protein synthesis required for CD8^+^ T cell activation (7), the UPR is well positioned to play a critical role in regulating T cell fitness and anti-tumor function. Consistent with this notion, IL-2 stimulation alone induces XBP1u upregulation, while TCR engagement promotes its splicing into the active XBP1s isoform (14). Although these findings establish a link between UPR activation and CD8^+^ T cell stimulation, the functional consequences of XBP1s signaling in this context remain incompletely defined. Previous studies have reported that the loss of XBP1s in CD8^+^ T cells does not alter peripheral CD8^+^ T cell frequency (13), nor does the loss of IRE1α affect T cell development (12). In contrast, cholesterol accumulation within the TME has been shown to induce XBP1s expression, resulting in increased PD-1 levels on CD8^+^ T cells (15). Although these findings suggest that there are context-dependent roles for UPR signaling in CD8^+^ T cells, critical gaps remain in our understanding of how sustained UPR activation influences CD8^+^ T cell homeostasis and anti-tumor immunity, as well as the immunologic and therapeutic consequences of targeting this pathway to modulate tumor burden.

A key innovation of this study is the development of a novel mouse model utilizing an E8_I_-CD8Cre-LSL system to specifically upregulate XBP1s in CD8^+^ T cells *in vivo*, enabling direct interrogation of the consequences of sustained UPR activation. In contrast to prior studies that have largely focused on loss-of-function approaches, the gain-of-function model permits detailed analysis of how chronic XBP1s signaling shapes CD8^+^ T cell homeostasis and function. Using this model, we found that constitutive UPR activation in homeostatic CD8^+^ T cells results in decreased cell numbers. Although this reduction may be driven by apoptosis induced by persistent UPR signaling, the underlying mechanisms remain to be fully defined and warrant further investigation, particularly given the established capacity of chronic UPR activation to trigger cell death through multiple pathways (47). Functionally, CD8^+^ T cells undergoing sustained UPR activation have reduced anti-tumor immunity, resulting in increased tumor burden that was associated with defective activation and migratory capacity. Notably, pharmacologic inhibition of IRE1α-mediated XBP1 splicing reduced tumor burden and was accompanied by a reduction in terminally exhausted CD8^+^ T cell populations and an expansion of more functionally competent, tumor-engaged T cell states in the TME. Together, these findings identify chronic UPR signaling as a previously underappreciated regulator of CD8^+^ T cell fitness and highlight therapeutic inhibition of the IRE1–XBP1 axis as a potential strategy to enhance anti-tumor immunity.

Future studies will be necessary to evaluate the therapeutic potential of combining B-I09 with immune checkpoint blockade (ICB). We speculate that such an approach could be synergistic, with B-I09 having a dual effect on both tumor cells and the immune compartment, while ICB boosts CD8^+^ T cell effector responses. Additional studies are also required to define the transcriptional networks downstream of sustained UPR activation that mediate these effects. Although prior studies have linked UPR signaling to T cell activation and expression of exhaustion-related markers, our data demonstrate that constitutive XBP1s activity alone is sufficient to compromise CD8^+^ T cell survival, activation, and migration capacity, ultimately limiting effective anti-tumor immunity. Mechanistically, our data point to the T-bet–CXCR3 axis as a key downstream mediator of XBP1s-driven functional impairment. XBP1s overexpression significantly reduced *Cxcr3* transcript levels, which was validated at the protein level (**Figure 4**), and was accompanied by decreased T-bet (*Tbx21*) expression in tumor-infiltrating CD8^+^ T cells (**Supplementary Figure S3**). As T-bet is a key transcriptional activator of CXCR3 expression (38), these data suggest that chronic XBP1s signaling may suppress the T-bet–CXCR3 migratory axis, thereby impairing T cell recruitment to the TME. Future experiments will be required to determine whether XBP1s directly regulates *Tbx21* transcription or acts through intermediary pathways to suppress T-bet activity. Further experiments will be required to determine whether XBP1s directly regulate *Tbx21* transcription or acts through intermediary pathways to suppress T-bet activity. We favor an indirect model in which XBP1s-driven suppression of T-bet secondarily limits CXCR3 and effector cytokine expression, though a direct effect of XBP1s at the *Cxcr3* or *Tbx21* loci cannot currently be excluded. Chromatin-binding assays (e.g., ChIP or CUT&RUN for XBP1s occupancy) and T-bet rescue experiments will be required to formally distinguish these possibilities. Interpretation of these results is tempered by the use of the E8_I_-CD8Cre system, which drives constitutive XBP1s expression from CD8 upregulation during thymic development and may introduce homeostatic confounders that obscure tumor-specific effects. Inducible or activation-restricted Cre systems (e.g., Granzyme B-Cre) would provide improved temporal control and more precisely define XBP1s function during anti-tumor effector responses. In addition, while the overexpression model supports a T cell–intrinsic role, pharmacologic inhibition in immunocompetent mice does not distinguish intrinsic from extrinsic effects. Approaches such as adoptive transfer into lymphopenic hosts (e.g., Rag^-^/^-^) or T cell–specific conditional knockout models will be important to resolve this.

We also note an apparent difference in outcome between our genetic and pharmacologic models: XBP1s-OE mice show reduced CD8^+^ T cell numbers in tumors and dLNs, whereas B-I09 restores CXCR3 expression without changing overall CD8^+^ T cell numbers. We attribute this to the differing timescales of the two interventions. XBP1s-OE imposes constitutive signaling from thymic CD8 upregulation onward, producing a chronically diminished peripheral CD8^+^ T cell pool prior to tumor challenge (**Figures 2D, E**), whereas B-I09 is administered for only two weeks in mice with an otherwise developmentally normal CD8^+^ T cell compartment, which was sufficient to restore CXCR3 expression on existing cells but not to substantially alter total cell numbers over this shorter window.

Similarly, our current data cannot formally establish that the anti-tumor effect of B-I09 is mediated primarily through CD8^+^ T cells, given that IRE1α/XBP1 signaling is also active in tumor cells and other immune populations. In our study, B-I09 reduced tumor burden without significantly increasing the frequency or absolute number of CD8^+^ or CD4^+^ T cells infiltrating the tumor or draining lymph node (**Figure 5B**), instead altering the differentiation and exhaustion state of the existing tumor-infiltrating CD8^+^ compartment (**Figures 5C–F**). This pattern, together with prior evidence that B-I09 has direct tumor-intrinsic activity in IRE1α/XBP1-dependent cancers independent of the immune system, is compatible with a model in which B-I09 acts, at least in part, directly on tumor cells, with a qualitative rather than quantitative effect on the CD8^+^ compartment. CD8^+^ T cell depletion or genetic ablation experiments combined with B-I09 treatment represent the definitive test of T cell dependence and are an important priority for future investigation. Collectively, these findings identify sustained UPR activation as a previously underappreciated intrinsic regulator of CD8^+^ T cell homeostasis and tumor-associated function and support UPR inhibition as a promising therapeutic strategy for the treatment of solid tumors.

## Data Availability

The RNA-sequencing data presented in the study have been deposited in the Gene Expression Omnibus (GEO) under accession number GSE346375.
