# Supplementary material for: Chronic XBP1s-mediated activation of the unfolded protein response impairs CD8+ T cell anti-tumor immunity
Source: Front Immunol. 2026 Oct 2;17:1940201. doi: 10.3389/fimmu.2026.1940201 (PMC13672639; doi:10.3389/fimmu.2026.1940201)
Supplement: Supplementary file 1 [file DataSheet1.pdf]

Figure S1

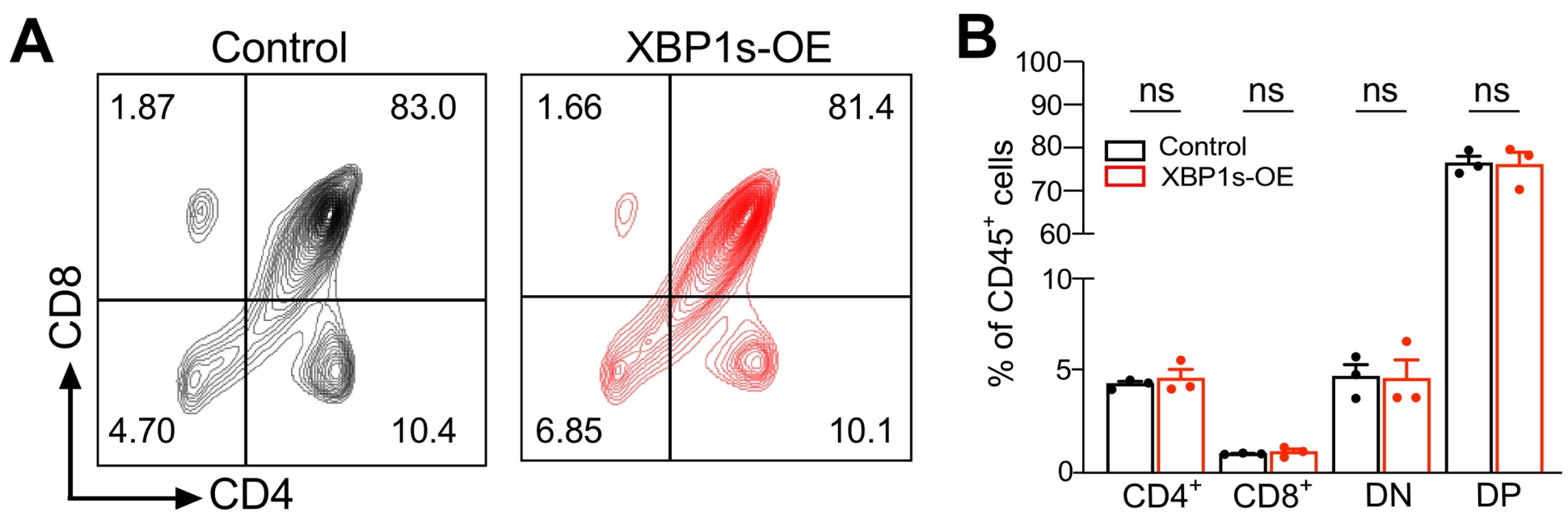

Figure S1. XBP1s overexpression does not affect thymic development. (**A**) Representative flow cytometry plots of thymocytes from control and XBP1s-OE mice. **(B)** Quantification of data from (A). Flow cytometry data are gated as follows: FSC-A/SSC-A, Live/Dead-negative, $CD45^+$. (n = 3 per group). Data are presented as mean ± SEM. ns: not significant. Statistical significance was assessed using a two-tailed unpaired t-test.

Figure S2

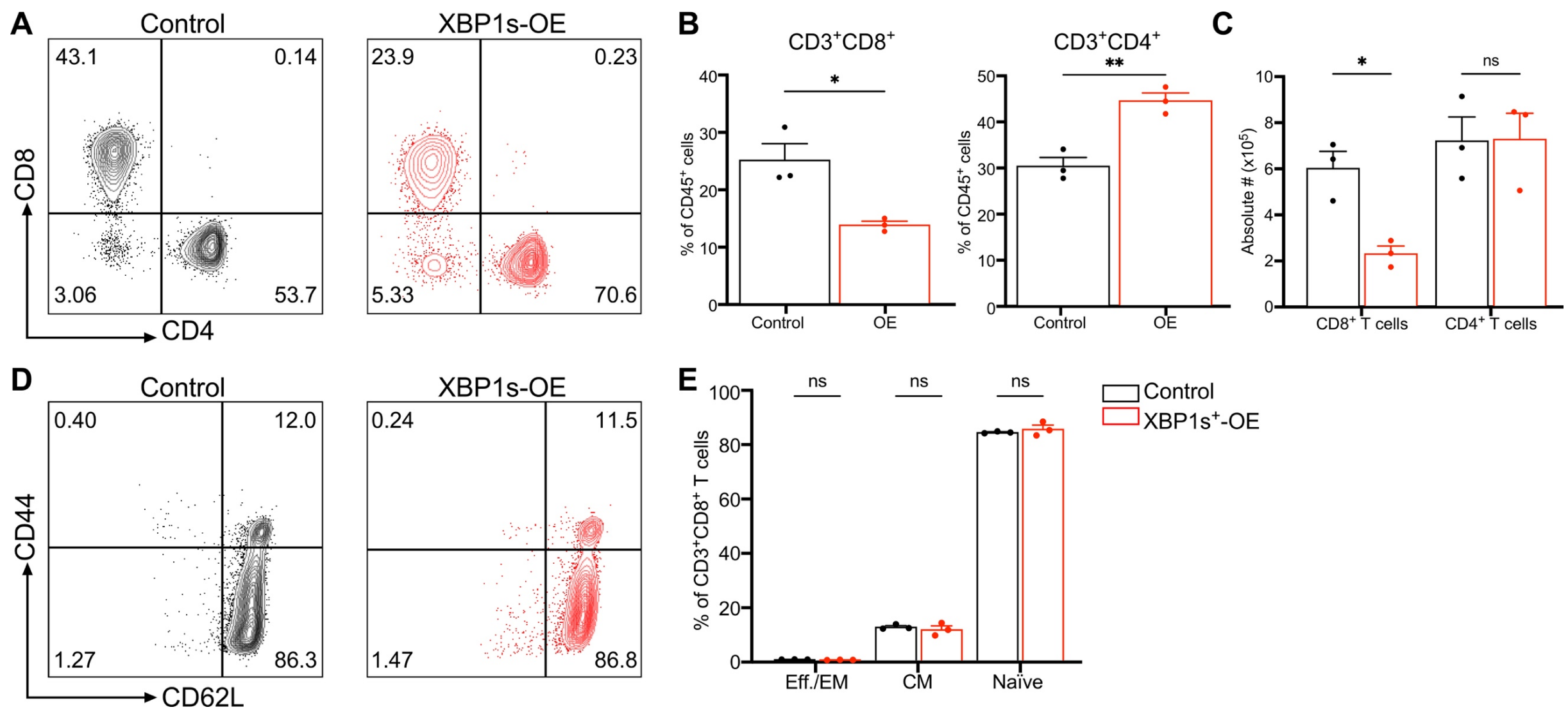

Figure S2. XBP1s overexpression decreases CD8⁺ T cell frequency in inguinal lymph nodes. (**A**) Representative flow cytometry plots of CD4⁺ and CD8⁺ T cells. (**B**) Quantification of T lymphocytes from control and XBP1s-OE mice. Flow cytometry data are gated as follows: FSC-A/SSC-A, Live/Dead-negative, CD45⁺, CD3⁺. (**C**) Absolute numbers of CD4⁺ and CD8⁺ T cells, calculated from total live cell counts. (**D**) Representative flow cytometry plots of CD62L and CD44 expression on T cells. (**E**) Quantification of T cell subsets. Flow cytometry data are gated as follows: FSC-A/SSC-A, Live/Dead-negative, CD45⁺, CD3⁺, CD8⁺, eGFP/XBP1s⁺. Data are presented as mean ± SEM, n = 3 mice per group. * $P < 0.05$, ** $P < 0.01$, *** $P < 0.001$, **** $P < 0.0001$. Statistical significance was assessed using a two-tailed unpaired t-test.

Figure S3

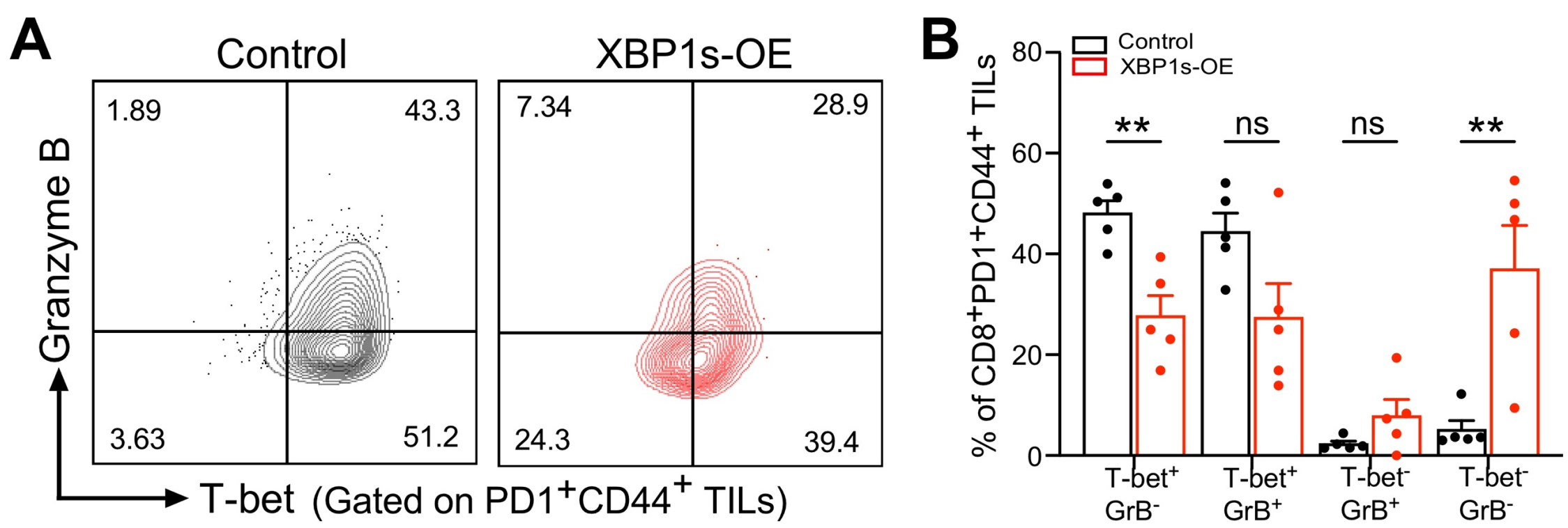

Figure S3. Overexpression of XBP1s reduces T-bet expression in the tumor microenvironment. (**A**) Representative flow cytometry analysis. (**B**) Quantification comparing PD1$^+$CD44$^+$ control and XBP1s-OE tumor-infiltrating lymphocytes. Data are presented as mean ± SEM, n=5 mice per group. * $P < 0.05$, ** $P < 0.01$, *** $P < 0.001$, **** $P < 0.0001$. Statistical significance was determined by a two-tailed unpaired t-test.

Figure S4

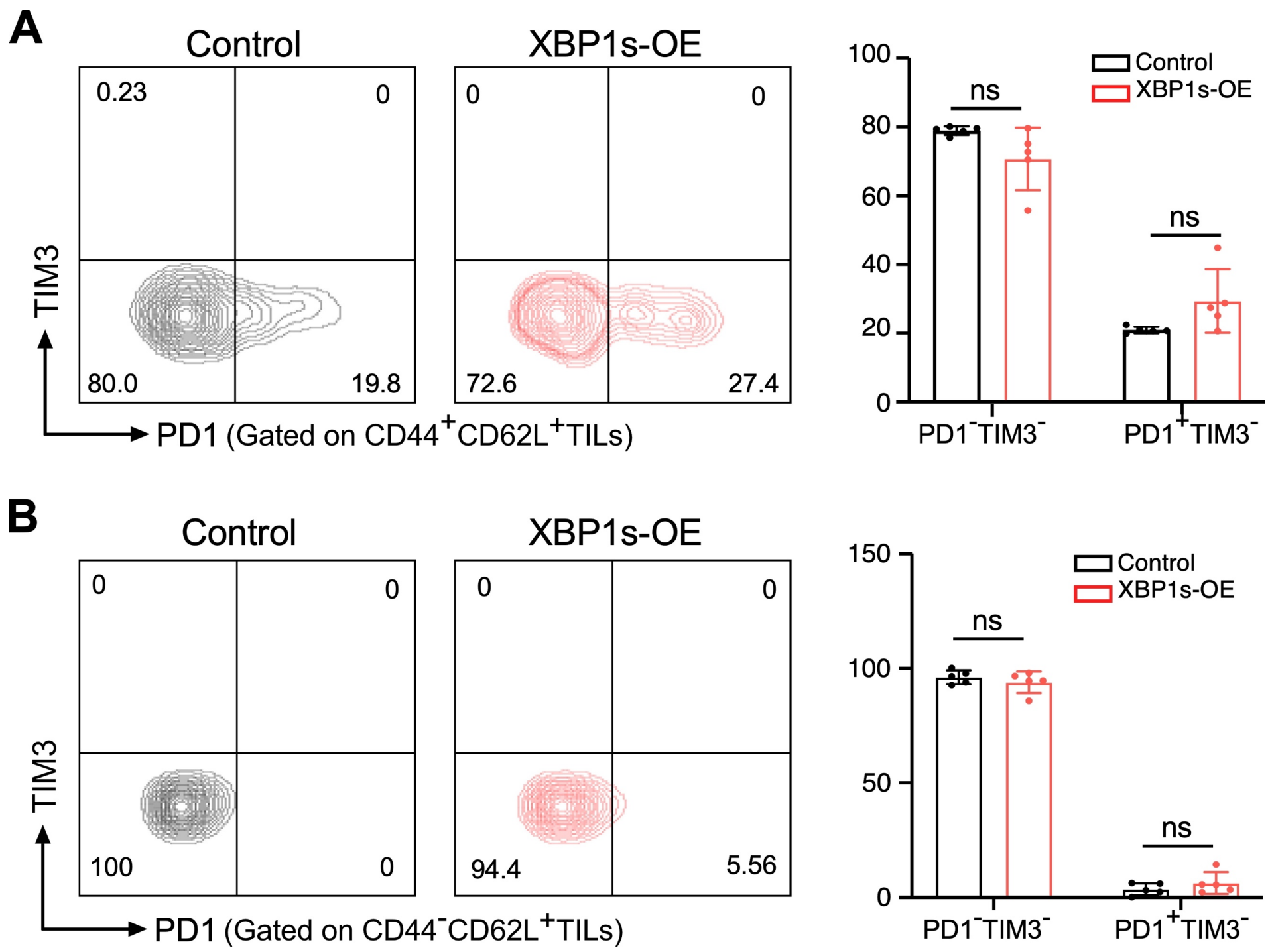

Figure S4. XBP1s overexpression does not affect PD1 and TIM3 expression levels on tumor-infiltrating central memory and naive CD8⁺ T cells. (**A**) Representative flow plots and quantification of PD-1 and TIM3 expression on $CD44^+$ $CD62L^+$ $CD8^+$ T cells isolated from tumors. (**B**) Representative flow plots and quantification of PD-1 and TIM3 expression on $CD44^-$ $CD62L^+$ $CD8^+$ T cells from tumors. Data are presented as mean ± SD, n=5 mice per group. ns: not significant. Statistical significance was determined by a two-tailed unpaired t-test.

Figure S5

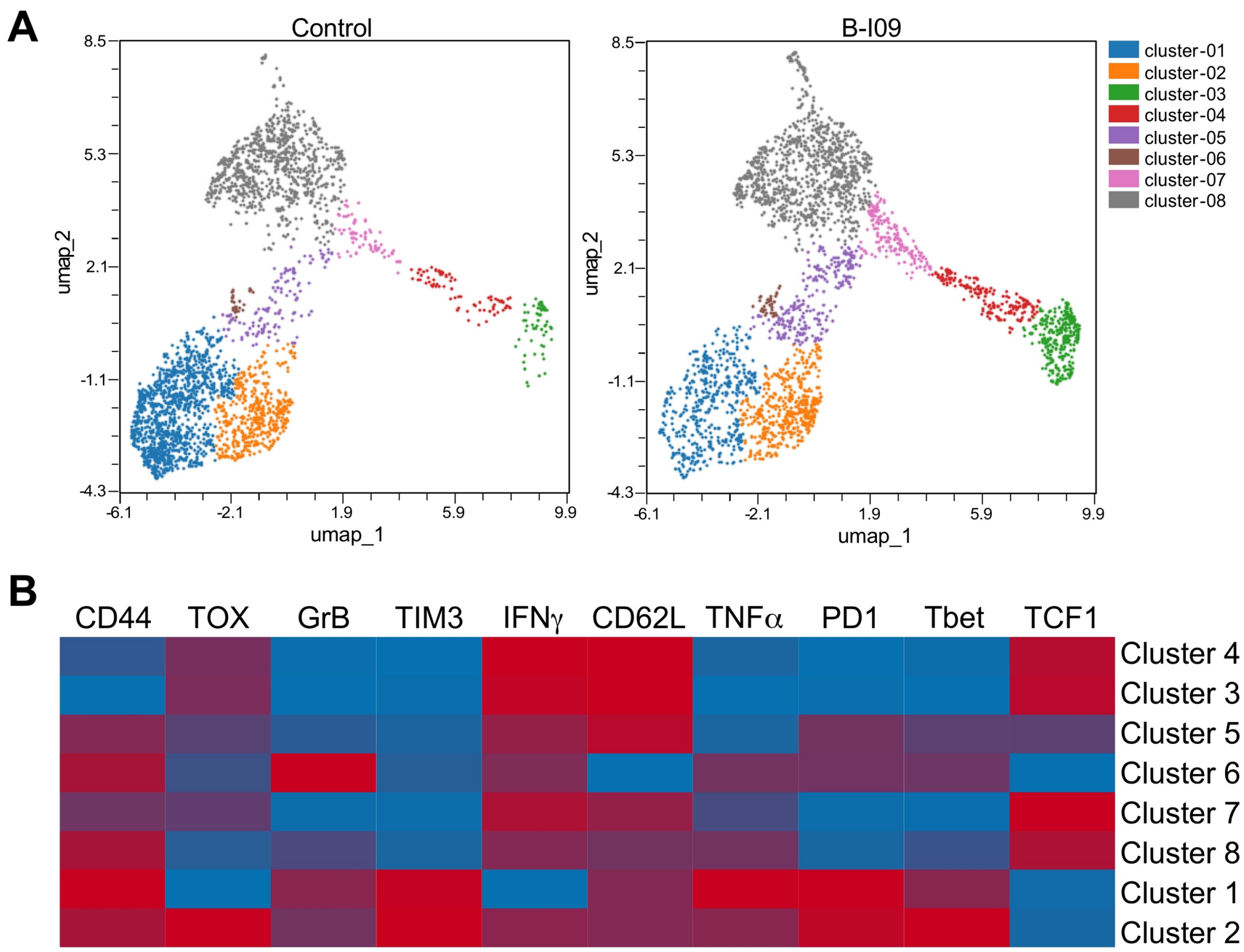

Figure S5. B-I09 treatment promotes an early-exhausted transcriptional phenotype. (**A**) Visualization of flow cytometry intracellular staining of $CD8^+$ T cells in the vehicle control and B-I09-treated mice. Graphs represent concatenated samples. n=3 per group. (**B**) Heatmap showing relative mean fluorescence intensity of intracellular marker expression in each cluster from panel A.
